# Supplementary material for: Improving the Comprehension of Pathogenicity and Phylogeny in ‘Candidatus Phytoplasma meliae’ through Genome Characterization
Source: Microorganisms. 2024 Jan 11;12(1):142. doi: 10.3390/microorganisms12010142 (PMC10819327; doi:10.3390/microorganisms12010142)
Supplement: Supplementary file 1 [file microorganisms-12-00142-s001.zip › Table S1.pdf]

Table S1: List of reference genomes used in this study. In bold draft genome obtained in this paper

Ca. Phytoplasma	strain	16Sr group/subgroup	Host	Country	accession	reference
Ca. P. asteris	AYWB	16SrI-B	*Lactuca sativa*	USA	CP000061.1	[26]
Ca. P. asteris	MBS	16SrI-B	*Zea mays*	Brazil	CP015149.1	[18]
Ca. P. asteris	OY-M	16SrI-A	*Chrysanthemum coronarium*	Japan	AP006628.2	[16]
Ca. P. australiense	PAa	16SrXII-B	*Catharanthus roseus*	Australia	AM422018.1	[31]
Ca. P. australiense	NZSb11	16SrXII-B	*Fragaria* x *anannassa*	New Zealand	CP002548.1	[40]
Ca. P. mali	AT	16SrX-A	*Catharanthus roseus*	Germany	CU469464.1	[41]
Ca. P. ziziphi	Jbw-nky	16SrV-B	*Ziziphus jujuba Mill.*	China	CP025121.1	[42]
Ca. P.s pruni	ChTDIII	16SrIII-B	*Melia azedarach*	Argentina	JABUOH000000000.1	[43]
Ca. P. solani	SA-1	16SrXII-A	*Vitis vinifera*	Italy	MPBG00000000.1	[20]
Ca. P. solani	231/09	16SrXII-A	*Petroselinum sativum*	Serbia	FO393428.1	[44]
Ca. P. solani	284/09	16SrXII-A	*Nicotiana tabacum*	Serbia	FO393427.1	[44]
Ca. P. australasiaticum	NTU2011	16SrII-V	*Arachis hypogaea*	Taiwan	NZ_AMWZ00000000.1	[17]
Ca. P. hispanicum	StrPh-CL	16SrXIII-F	*Fragaria* x *anannassa*	Chile	JAGVRH000000000.1	[30]
***Ca*. P. meliae**	**ChTYXIII**	**1SrXIII-G**	***Melia azedarach***	**Argentina**	**NZ_JACAOD000000000.2**	**This paper**
