# Supplementary figures and images for: Improving the Comprehension of Pathogenicity and Phylogeny in ‘Candidatus Phytoplasma meliae’ through Genome Characterization
Source: Microorganisms. 2024 Jan 11;12(1):142. doi: 10.3390/microorganisms12010142 (PMC10819327; doi:10.3390/microorganisms12010142)

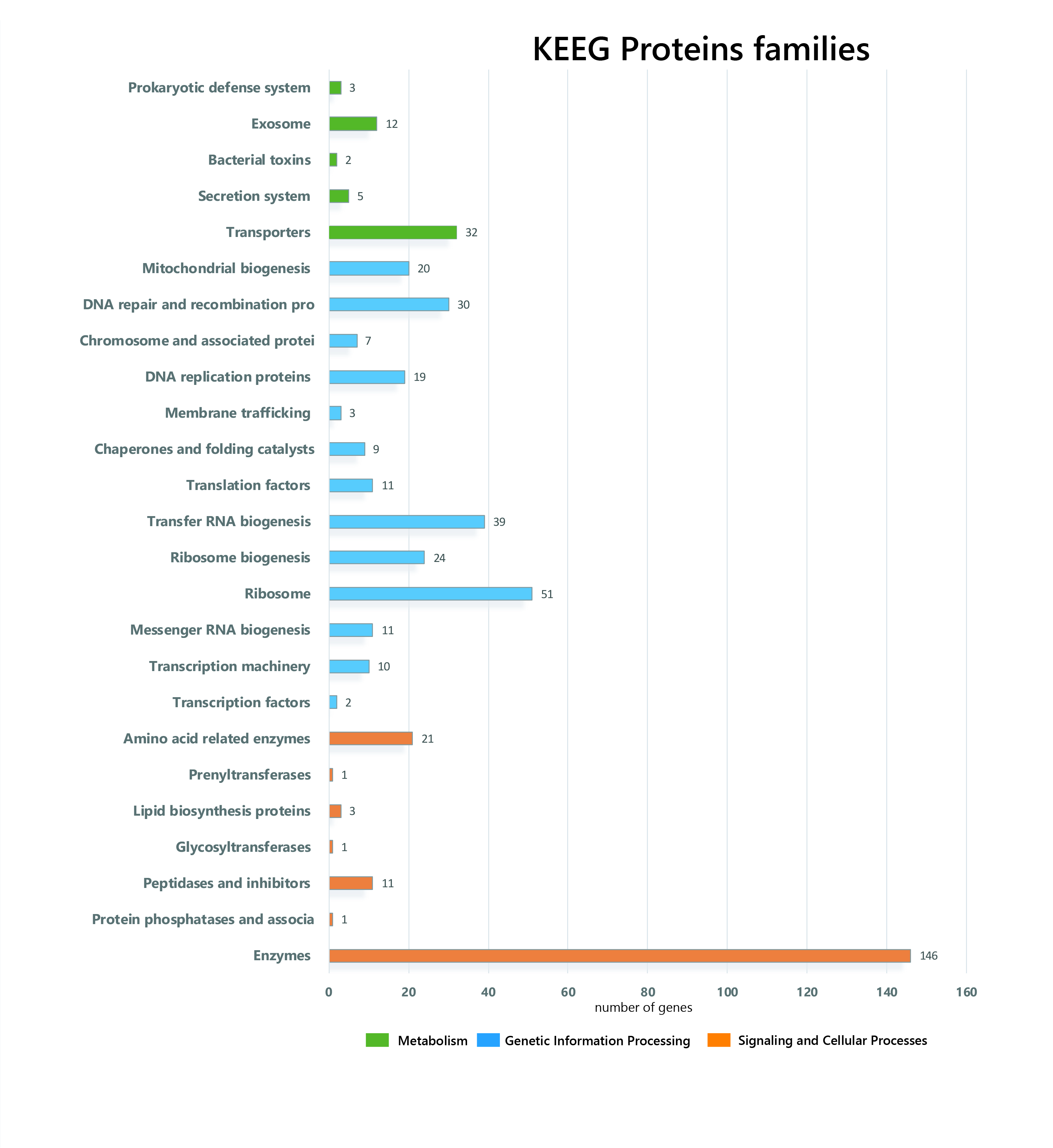

Supplement: Supplementary file 1 [file microorganisms-12-00142-s001.zip › Figure_S1 v15-12-2023.tif]

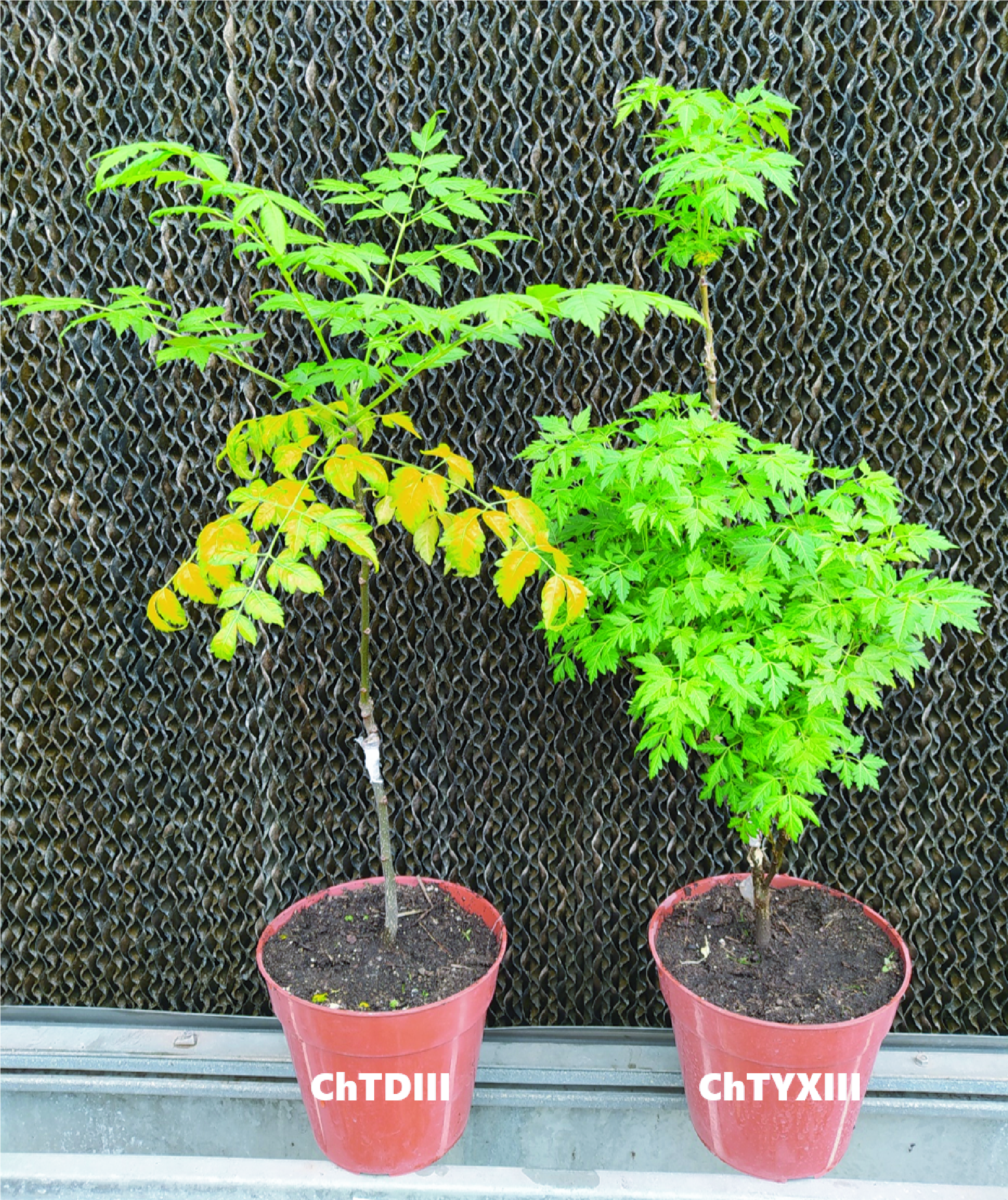

Supplement: Supplementary file 1 [file microorganisms-12-00142-s001.zip › Figure_S2 v15-12-2023.tif]

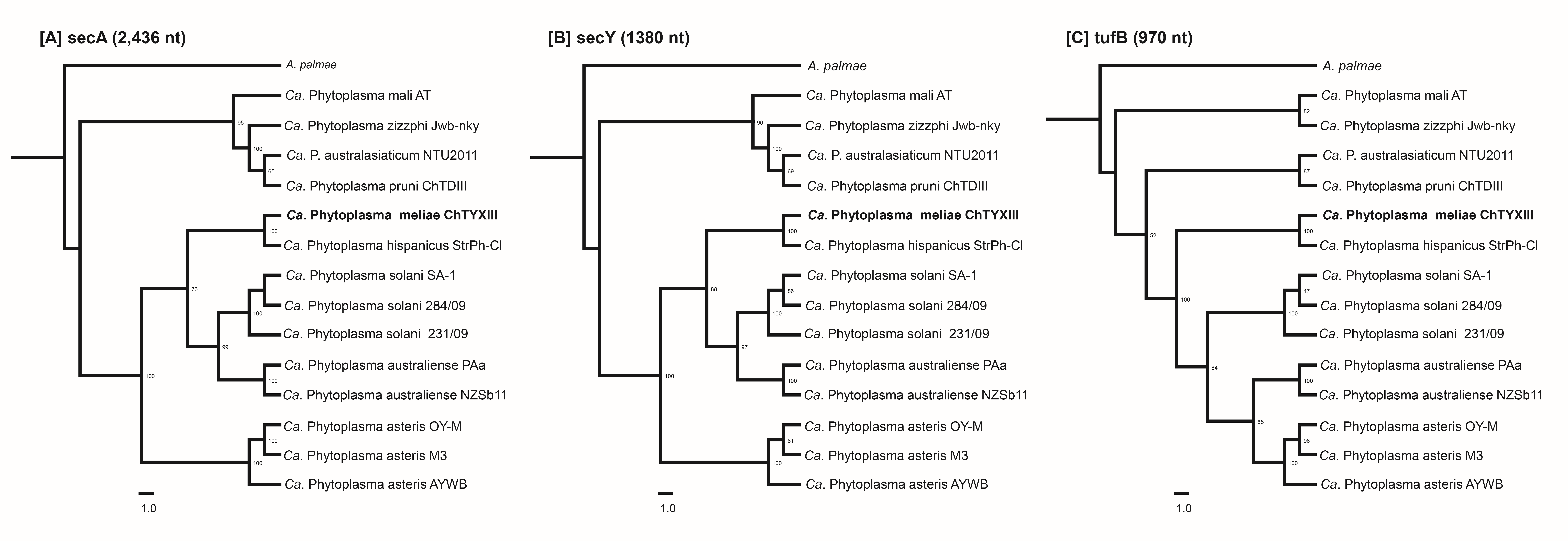

Supplement: Supplementary file 1 [file microorganisms-12-00142-s001.zip › Figure_S3 v18-12-2023.tif]
